# Supplementary material for: Elucidating the Local Transmission Dynamics of Highly Pathogenic Avian Influenza H5N6 in the Republic of Korea by Integrating Phylogenetic Information
Source: Pathogens. 2021 Jun 2;10(6):691. doi: 10.3390/pathogens10060691 (PMC8230294; doi:10.3390/pathogens10060691)
Supplement: Supplementary file 1 [file pathogens-10-00691-s001.zip › pathogens-1228273-supplementary.pdf]

Supplementary material

Materials and methods

Table S1. Poultry holdings classification for likelihood function for transmission kernel parameter estimation.

Classification (prevalent genotype)	Cluster A (C3)	Cluster B (C4)
Total no. poultry farms	680	160
No. uninfected farms (K group)	363	8
No. PCs without infection (F group)	259	104
No. IPs with different genotype (D group)	16	0
No. IPs with the same genotype (M group)	42	48

IPs, infected premises; PCs preemptive culled premises

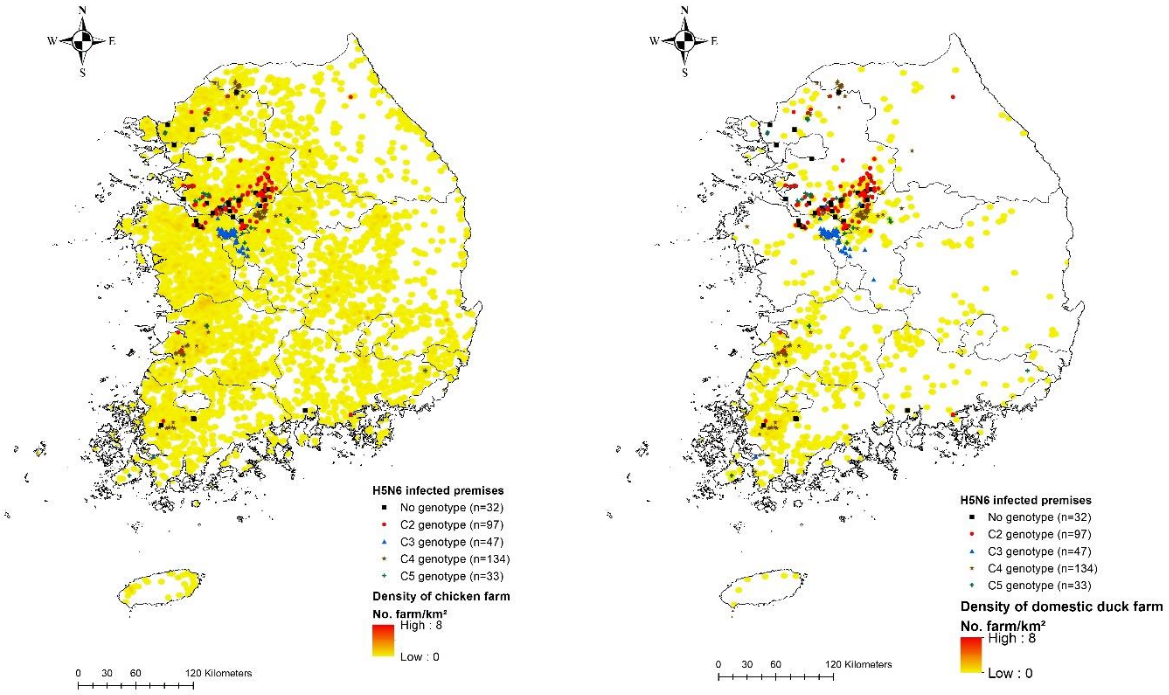

Figure S1. The geographical distribution of domestic chicken farms (left) and domestic duck farms (right). The bright yellow shading represented low density of poultry farms, wherease red shading denoted high density of poultry farms. Red dots, blue triangles, brown stars, and green crosses denote premises infected with the C2-genotype virus, C3 virus, C4 virus, and C5 virus, respectively. Gray dots indicate all other poultry holdings.

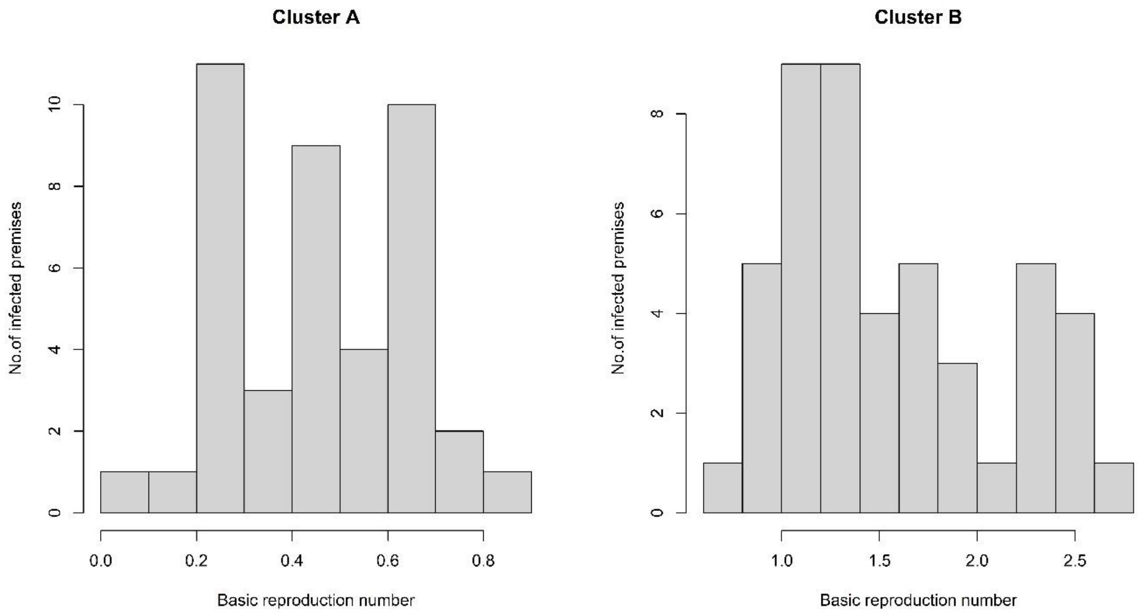

Figure S2. The distribution of the basic reproduction number of IPs in two spatio-temporal clusters for HPAIv H5N6 outbreaks.

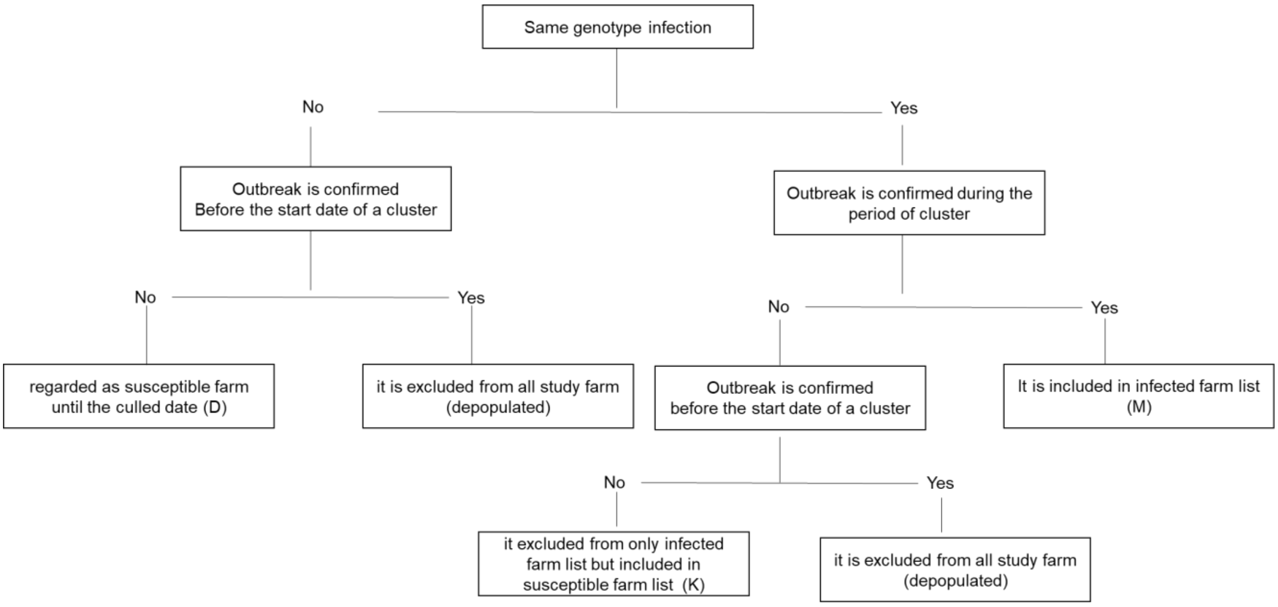

Figure S3. Schematic representation of classification trees for HPAI infected premises. We excluded the farms that stop their production or culled before the start time of each cluster. For example, if HPAI H5N6 C2 genotype infection was significantly spatial clustered from November 26th, 2016 to January 6th, 2017, IP A that had HPAI H5N6 C2- genotype infection on November 18th, 2016, and depopulated on November 19th, 2016, was excluded from the list of study farms. In contrast, if the IP had the C2-genotype of infection on January 10th, 2017, placed in the C2-genotype cluster, it was included and regarded as the susceptible state during times specified by spatiotemporal clusters. This process holds for different genotype infected premises because they would be susceptible farms at risk who are subjected to receive the force of infection before the observed infection date.

Results

Table S2. Sensitivity analysis of various infectious duration assumption of infected premises on transmission kernel parameter estimates.

Category	Infectious periods	h_0	r_0	α	AIC
	7days	0.00062	2.6030	1.363	633.99
Cluster A	14days	0.00019	6.0781	1.3306	638.60
	21days	0.00007	6.6906	1.2684	668.36
	7days	0.00262	2.2460	1.3580	483.64
Cluster B	14days	0.00146	1.2225	1.3775	550.00
	21days	0.00122	1.1670	1.3785	538.77

AIC, Akaike Information Criterion

Table S3. Summary of the simulation outcomes for different preemptive culling radii from HPAI infected premises.

Culling radius	Cluster A		Cluster B	
	No. IPs median (5-95 percentile)	No. PCs median (5-95 percentile)	No. IPs median (5-95 percentile)	No. PCs median (5-95 percentile)
0.5km	18 (8,31)	578 (366, 646)	67 (41, 92)	103 (80, 125)
1km	18 (8,30)	575 (387, 644)	59 (37, 83)	111 (87, 129)
2km	18 (10,31)	576 (383, 645)	54 (31,80)	115 (93, 133)
3km	17 (8, 30)	609 (535, 636)	52 (29, 77)	118 (94, 136)
Observed (3km)	42	259	48	104

IPs, infected premises; PCs preemptive culled premises. The number of IPs and PCs presented in the table corresponded to the cumulative median number of cases and depopulated farms during the average extinction days of 1,000 iterations.
